# Impact of the COVID-19 Pandemic on HPV Vaccinations in Switzerland and Greece: Road to Recovery

**DOI:** 10.3390/vaccines11020258

**Published:** 2023-01-25

**Authors:** Ilias Gountas, Andrea Favre-Bulle, Kunal Saxena, Jessica Wilcock, Hannah Collings, Stina Salomonsson, Anastasios Skroumpelos, Ugne Sabale

**Affiliations:** 1 MSD Greece, 17456 Alimos, Greece; 2 MSD Switzerland, CH-6005 Lucerne, Switzerland; 3 Center for Observational and Real-World Evidence (CORE), Merck & Co., Inc., Rahway, NJ 07065, USA; 4 Adelphi Values PROVE, Bollington SK10 5JB, UK; 5 Center for Observational and Real-World Evidence (CORE), MSD, 11330 Stockholm, Sweden

**Keywords:** HPV, routine vaccines, adolescents, COVID-19, Switzerland, Greece

## Abstract

The COVID-19 pandemic has caused significant disruptions to healthcare, including reduced administration of routinely recommended HPV vaccines in a number of European countries. Because the extent and trends of accumulated vaccine dose deficits may vary by country, decision-makers need country-specific information regarding vaccine deficits to plan effective catch-up initiatives. To address this knowledge gap in Switzerland and Greece, this study used a previously published COVID-19 recovery calculator and historical vaccine sales data to quantify the cumulative number of missed doses and the catch-up rate required to clear the deficit in Switzerland and Greece. The resultant cumulative deficit in HPV doses for Switzerland and Greece were 24.4% and 21.7%, respectively, of the total number of doses disseminated in 2019. To clear the dose deficit by December 2025, monthly vaccination rates must be increased by 6.3% and 6.0% compared to 2019 rates in Switzerland and Greece, respectively. This study demonstrates that administration rates of routine HPV vaccines decreased significantly among Swiss and Greek adolescents during the COVID-19 pandemic and that a sustained increase in vaccination rates is necessary to recover the HPV dose deficits identified and to prevent long-term public health consequences.

## 1. Introduction

Human papillomavirus (HPV) infection is the most frequent sexually transmitted disease and the second most common cause of cancer attributable to an infectious agent globally [1]. During the last 15 years, vaccines have been available to prevent HPV infection and HPV-associated illnesses [2,3,4]. Thus, HPV vaccination was introduced across Europe and is now routinely recommended for adolescent girls and boys in most European countries [5,6].

Since the outbreak of the SARS-CoV-2 virus and the subsequent COVID-19 pandemic declared in March 2020 [7], significant reductions in the administration of routine vaccines has been observed globally [8,9]. According to the World Health Organization (WHO), the substantial disruptions to routine pediatric immunization programs have affected more than 80 million children worldwide [10,11,12]. In particular, this disruption has largely affected adolescent vaccinations as a result of school closures and hesitancy to seek healthcare services as well as the diversion of time, resourcing, and funding to COVID-19 strategies within healthcare infrastructures [13,14,15,16].

Interruptions to scheduled immunizations and missed vaccinations, even for short periods, increase the number of susceptible individuals and raises the risk for outbreaks of vaccine-preventable diseases [11,12,13,14,15,16,17]. Health authorities have raised concerns over future public health implications caused by COVID-19-related routine vaccination gaps [9]. The WHO issued guidance encouraging prioritization of immunization and motivating countries to regularly evaluate the necessity for mass catch-up vaccination campaigns [18]. However, because the extent and trends of vaccine deficits may vary by country, decision-makers need country-specific, evidence-based information regarding vaccine deficits to plan accurate and effective catch-up initiatives [9].

Similar to other routine vaccinations, the HPV vaccination programs faced significant COVID-19-related interruptions, thus resulting in a substantial number of adolescents missing an opportunity to get vaccinated [17,19]. Consequences of missed HPV vaccinations include an excess of preventable cases of genital warts, cervical cancer, and other HPV-related diseases [11,17]. A modelling study examining the impact of first-wave COVID-19 measures in the USA reported a 75% reduction in HPV vaccination coverage in April 2020 compared to previous years, and warned that without significant public health interventions, a considerable increase in HPV-related diseases would be observed [17].

Although a slow recovery of vaccination rates has been observed globally, the vaccination gap accumulated during the COVID-19 pandemic persists in a number of European countries. Switzerland and Greece are two of the European countries where a reduction in the number of HPV doses administered during the pandemic was observed [20], albeit to differing extents across the two countries. Moreover, country-specific estimates of differently accumulated deficits in Switzerland and Greece will provide vital support to local decision-making on catch-up activities.

In Switzerland, the HPV vaccine is fully funded for both boys and girls aged 11–14 years old (primary cohort) and for 15–26 years old (catch-up, female patients only in 19–26 years old group) [21,22]. In Greece, the HPV vaccination program was targeted at 11–13-year-old girls (primary cohort) until March 2022, when a gender-neutral vaccination was introduced [23].

The aim of this study is to describe the COVID-19 related HPV vaccine deficit accumulation and to estimate the magnitude and duration of future catch-up required to clear the deficit in Switzerland and Greece.

## 2. Materials and Methods

### 2.1. Target Population

#### 2.1.1. Switzerland

As gender neutral HPV vaccination is included in the Swiss National Immunization Program, the target population in Switzerland for our analysis comprised adolescent boys and girls.

#### 2.1.2. Greece

Because a girls-only program was included in the Greek National Immunization program between 2019 and 2021, the studied time period in our analysis, the target population in Greece comprised adolescent girls.

### 2.2. Data Sources

HPV vaccine sales data were used as a proxy to inform the number of HPV vaccine doses administered. For the purpose of this analysis, HPV vaccine doses sold are referred as HPV vaccine doses administered.

For Switzerland, the monthly number of HPV vaccine doses between January 2019 and December 2021 were sourced from the IQVIA Database [24]. The IQVIA sales database is a well-established data source that provides validated real-world sales data used in a wide range of countries where alternative data sources are limited [25]. For Greece, monthly vaccine sales data for the period January 2019–December 2021 were used (data on file) (Table S1).

### 2.3. The COVID-19 Recovery Tool

To quantify the projected cumulative number of HPV doses missed and to estimate the catch-up vaccination rate required to clear the HPV vaccination deficit, a previously published Excel-based COVID-19 recovery tool was used [19]. Although this tool was originally developed for a US setting, no adaptations to the model structure were required for the Greek and Swiss settings in this study, only to the model inputs.

The tool describes the course of the HPV vaccination deficit through three sequential periods: the COVID-19 impact period, the transition period, and the catch-up period. The COVID-19 impact period is defined as the period when the administration of the HPV vaccine was impacted by the COVID-19 pandemic. The transition period lasts between the end of the COVID-19 period and the start of the catch-up period, in which the percentage change in vaccination uptake gradually increases. Finally, the catch-up period will be when monthly vaccination uptake rates are higher relative to the corresponding month in the reference period. Model inputs reflect each country-specific setting and therefore lead to differences in the durations of the modeled time periods between countries.

Inputs to the model included (i) observed monthly vaccine administration data before and during the COVID-19 pandemic, (ii) the start date of the transition period, and (iii) the start of the catch-up period.

**Figure 1:**
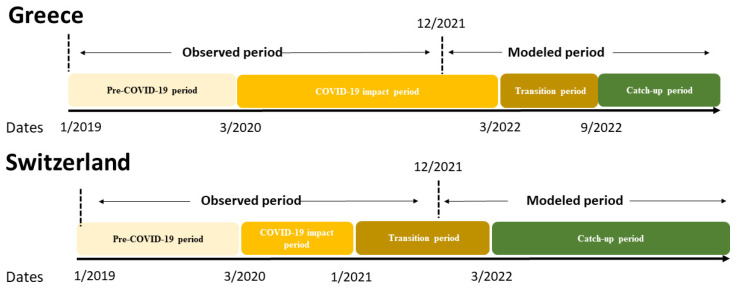
Schematic outline of the analysis for each country.

The model outputs included the cumulative deficit in doses administered, estimates for time required to catch up, and catch-up rates needed to recover from the deficits. The modelled periods for each country are presented in Figure 1 and Table S1.

### 2.4. Estimating the HPV Dose Deficit

During the observed period, the dose deficit was estimated by subtracting the number of monthly doses administered during the COVID-19 pandemic (i.e., years 2020 and 2021) from the number of doses administered in the corresponding month of the pre-pandemic year (2019).

For Greece, where the COVID-19 impact period lasted more than the observed period, the missing data (i.e., 3 months) were imputed. Imputation was calculated by summing the monthly doses administered in the years 2019 (the last pre-pandemic year) and 2021 to estimate the relative yearly difference in administered doses (Figure S2), and then multiplying the relative yearly difference by the corresponding doses administered in each month of 2019.

### 2.5. Examined Scenarios

To illustrate the magnitude and duration of future catch-up efforts required to clear the deficit in missed HPV doses due to the COVID-19 pandemic, three scenarios were considered: a base case, an optimistic scenario, and a pessimistic scenario. The base case examined the required catch-up rate to clear the effect of the COVID-19 pandemic by the end of 2024, whereas the optimistic and pessimistic scenarios explored the required catch-up rates to clear the deficit by the end of 2023, or 2025, respectively.

## 3. Results

### 3.1. HPV Vaccination Deficit Accumulation

To characterize the course of the deficit caused by the pandemic, both the duration and the size of the deficit were described.

#### 3.1.1. Switzerland

**Figure 2:**
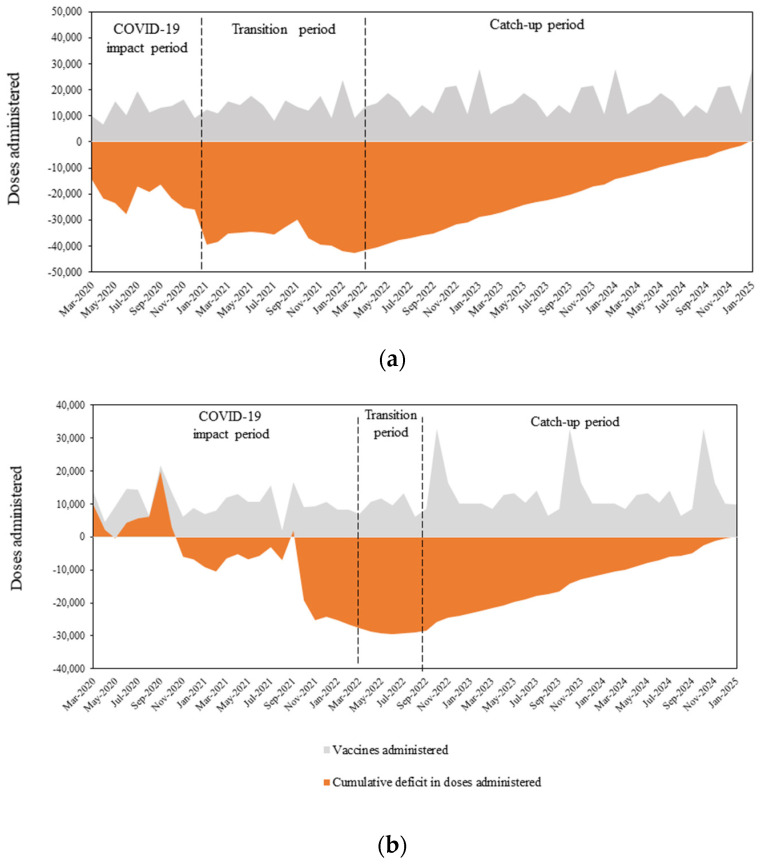
Deficits in vaccine doses administered due to the COVID-19 pandemic and model predictions for clearing the cumulative HPV deficit by the end of 2024 in (**a**) Switzerland and (**b**) Greece.

During 2020, HPV vaccine administration was reduced by 14.8% compared to 2019 (Figure S1). In the second year of the pandemic, the relative reduction in the number of vaccine doses disseminated was lower (7.9%), indicating that a recovery in Switzerland had already begun in 2021, as the dose deficit in 2021 decreased by 46.6% compared to 2020 (Figure S1). The observed data show that the dose deficit was accumulated over 9 months (Figure 2).

#### 3.1.2. Greece

As the first COVID-19 wave was relatively mild in Greece [26], the deficit in HPV vaccinations started to accumulate later compared to Switzerland (Figure S1). Specifically, the HPV vaccination deficit started to accumulate at the start of the second wave of the COVID-19 pandemic. The dose deficit in 2021 increased by 156.2% compared to 2020 (Figure S1). Since the transition period began in March 2022, the HPV vaccine dose deficit accumulated for 23 months (Figure 2). The percentage reduction in annual HPV doses administered during 2020 and 2021 compared to 2019 were 4.8% and 12.3%, respectively.

These results demonstrate that HPV vaccination was affected differently in Switzerland and Greece and thus highlight the need for country-specific decisions on how to clear the accumulated dose deficits.

### 3.2. Cumulative Deficit of HPV Vaccine Doses

The dose deficit accumulated both at the start of the transition period and the start of the catch-up period is shown in Table 1. For Switzerland, the estimated cumulative HPV dose deficit at the start of the catch-up period was 42,680 doses, which corresponds to 24.4% of the total number of doses disseminated in 2019, or 2.9 months of vaccination in 2019. For Greece, the estimated cumulative HPV dose deficit at the start of the catch-up period was 31,000 doses. This corresponds to 21.7% of the total doses administered in 2019, or 2.6 months of vaccination in 2019 (Table 1).

### 3.3. Catch-Up Rate Scenarios

In the base case scenario, to clear the dose deficit by December 2024, monthly vaccination would need to be increased by 8.4% (1226 additional doses/month) and 8.2% (975 additional doses/month) compared to 2019 rates in Switzerland and Greece, respectively (Table 2, Figure 2).

Higher clearance was modelled in the optimistic scenario, wherein monthly vaccination catch-up rate increases of 12.6% and 13.0% in Switzerland and Greece would clear the deficit by the end of 2023. The pessimistic scenario, which assumed the dose deficit would be cleared by the end of 2025, required catch-up rate increases of 6.3% and 6.0% in Switzerland and Greece, respectively (Table 2, Figures S2 and S3).

**Figure 3:**
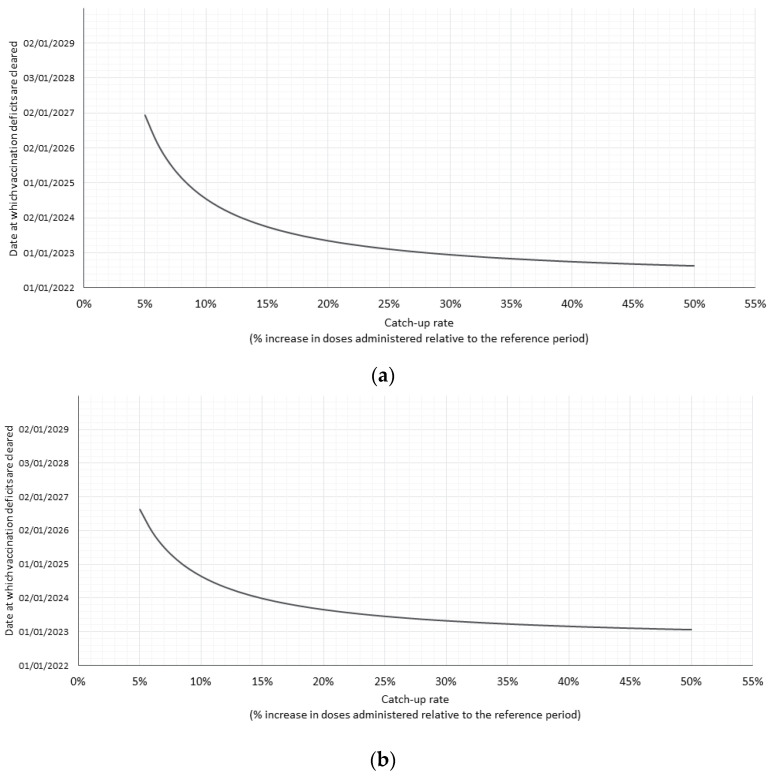
Date at which cumulative missed HPV doses reaches zero according to catch-up rate in (**a**) Switzerland and (**b**) Greece.

The relationship between catch-up rates and the time required to clear cumulative HPV dose deficits is shown in Figure 3. In both countries, a 5% catch-up rate would be sufficient to clear the effect of the pandemic by 2027.

## 4. Discussion

The COVID-19 pandemic significantly affected the administration of routine vaccines globally [8,9,27]. Our analysis assessed the impact of the COVID-19 pandemic on the cumulative number of missed HPV vaccine doses and the requirements to clear the backlog of HPV vaccines. It was demonstrated that despite country-specific differences, the projected cumulative deficit of missed doses was substantial in both countries (21.7% and 24.4% in Greece and Switzerland, respectively), and an increase between 6% and 13% in 2019 vaccination rates will be required to clear the dose deficit between the end of 2023 and the end of 2025.

Our findings add to a growing body of evidence showing considerable decreases in the administration of adolescent vaccines in the US and globally as a result of the COVID-19 pandemic [9,17,19,28]. In France, a 33% reduction in HPV vaccinations was reported in 2020, and a 27% reduction was reported over the first four months of 2021 [29]. In Italy, an estimated 42% of girls and 52% of boys born between 2005 and 2009 were not vaccinated against HPV up to 2021 [30]. As missed HPV vaccinations would result in an increase in HPV-related morbidity and mortality, timely catch-up initiatives are crucial for successful immunization program recovery and HPV prevention [17]. The speed and magnitude of catch-up programs will not only impact the number of individuals remaining susceptible to HPV, but also impact future health resources required to manage preventable HPV-related conditions [30]. For instance, a recent study showed that failure to clear the HPV dose deficit accumulated due to the COVID-19 pandemic in Italy would result in 1.1–1.3 million Italian adolescents not being protected against HPV-related diseases over their lifetime, with an associated cost of €905 million [30].

This analysis highlights that the COVID-19 pandemic affected the HPV immunization programs of the examined countries differently. Although Greece was not heavily impacted during the first half of the first year of the pandemic, the dose deficit started to accumulate in the second half of the first year. This could be partially explained by the severity of the COVID-19 infection waves (the subsequent waves were more severe than the first one) and the fact that a vaccine delivery system driven by pediatricians is less susceptible to external crises than a school-based vaccine delivery system. Although the HPV dose deficit began to accumulate later, Greece faced a longer HPV vaccine dose deficit accumulation period compared to Switzerland. In Switzerland, on the other hand, the largest HPV dose deficit was accumulated in 2020 and could be attributed to school closures and limited access to routine pediatrician consultations and general health services between March and July 2020. In 2021, certain policy measures were taken by the Swiss Ministry of Health and the National Immunization Technical Advisory Group to continue with routine immunization despite the pandemic. This resulted in Switzerland launching HPV deficit recovery by 2021 [21,31].

### 4.1. Study Implications

To optimize the effectiveness of HPV vaccination catch-up programs, they should be tailored to country-specific settings as well as health care and vaccine delivery systems. For instance, in settings with school-based vaccination programs, it is important to implement catch-up programs at school, a setting where it is easiest to reach the target population [32]. On the contrary, for countries where vaccination is primarily driven by general practitioners or pediatricians, initiatives targeted at both healthcare professionals and parents are preferred [33]. Digital reminder notifications, vaccination open-days, communication campaigns to raise awareness of negative consequences associated with vaccination gaps, and education programs for both physicians and parents are some of the best practices that have been implemented internationally in attempts to increase the rate of catch-up vaccination [34,35]. To catch-up unvaccinated individuals in a timely manner, novel approaches such as e-health could be utilized [34,35]. However, it is worth emphasizing that for effective monitoring of catch-up vaccination initiatives, continuous surveillance of vaccine coverage rates across European countries is essential [36].

Our analysis shows that both the magnitude and the duration of catch-up vaccinations are predominant factors in how soon the accumulated dose deficit can be cleared. As such, it is important to consider the age range of a publicly funded HPV vaccination program when designing effective catch-up initiatives. This is of particular importance and urgency for adolescents and young adults nearing the upper bound of the recommended vaccination age range, as these individuals may soon no longer be eligible for HPV vaccination [19]. Additionally, for adolescents over the age of 15, an additional dose will be required, which will further deepen the dose deficit if not recovered in time. The deficit may become even more profound if an individual will be required to pay out-of-pocket for the missed dose as a result of reaching a defined age-limit for publicly funded HPV vaccination.

Duration of a catch-up period is another important factor to be considered when planning effective recovery. For example, in countries such as Greece, where the announced national HPV catch-up program will end in December 2023 [37], a catch-up program must be either intensive or time-extended to ensure recovery. However, catch-up interventions should be as short-term as possible because the effectiveness of the intervention observed in these programs decreases with extended duration [38].

Differences in the accumulated HPV dose deficit between countries could be explained by the differences in healthcare systems, the timing of recovery initiatives, and the baseline vaccination rate. For example, in countries with a school-based vaccination system, the deficit will increase faster compared to countries without school-based vaccination programs [32]. On the other hand, school-based vaccination programs are found to be one of the most effective ways to improve vaccination coverage and, hence, may be a preferred platform for catch-up initiatives [39]. Countries with higher baseline rates are expected to be more substantially impacted by the COVID-19 pandemic than countries with lower vaccine coverage rates. Nevertheless, in countries with suboptimal vaccine coverage like Greece, the disruption during the COVID-19 pandemic further highlights the urgency to clear the dose deficit and continue improving vaccine coverage rates. Finally, the absolute HPV vaccination dose deficit is a function of the targeted population (e.g., the size of the population and whether the deficit was accumulated by all genders or by female patients only). Hence, for the reasons highlighted above, any comparison between countries should be made with caution.

Since its onset, the COVID-19 pandemic has significantly impacted essential health services, revealing the vulnerabilities of healthcare infrastructure [40]. To increase immunization programs’ resilience, countries should invest in strengthening national immunization infrastructures for both routine and emergency vaccinations [11]. For example, a significant proportion of existing vaccine centers are expected to be dedicated to COVID-19 vaccination; therefore, investing in resilient healthcare infrastructure is vital to minimize routine vaccination interruptions. Additionally, innovative approaches such as drive-thru clinics, as were implemented in the UK, may provide interesting solutions [41].

In 2020, the WHO launched the Global Strategy to Accelerate the Elimination of Cervical Cancer, with a key target of the initiative being 90% of girls receiving a full course of the HPV vaccine by the age of 15 years of age [42,43]. However, due to the pandemic, healthcare systems have deprioritized their HPV vaccine coverage improvement efforts [11]. This deprioritization will endanger the ambitious goal of cervical cancer elimination and create a generation of adolescents susceptible to HPV-related cancers. The Strategic Advisory Group of Experts on Immunization noted with alarm that HPV vaccination implementation is off-track to meet the elimination targets. Therefore, urgent post-pandemic actions should be taken to return and stay on track towards achieving the goal of having 90% of girls up to 15 years of age fully vaccinated against HPV by 2030.

### 4.2. Strengths and Limitations

The strength of our analysis is the combination of both real-world data from two different international settings and the use of a recently published modelling tool [19]. A potential source of bias in our study is that the input data used relied upon sales data, which may not take into account any potential stockpiled vaccines or vaccines administered in different health care sectors. The sales data used in this analysis primarily represent vaccine sales as part of the national immunization programs (NIP), wherein at the time of the analysis, a nine-valent HPV vaccine was used exclusively in both Swiss and Greek NIPs, providing a good overview of vaccine uptake. However, it is possible that a small proportion of doses were administered in a private market and to individuals in other cohorts.

As a modelled estimate, this analysis is not without limitations. First, the tool does not incorporate demographics (e.g., population growth and mortality rates), potentially underestimating vaccine deficits and, therefore, the necessary catch-up efforts required. Second, during the COVID-19 impact period, missing data for Greece were imputed using a simple methodology. However, the effect of this imputation is likely to be marginal because most of the data used to calculate the pandemic impact on HPV vaccinations were observed. Finally, the recommended vaccination schedule for HPV indicates that individuals are eligible for multiple doses during adolescent years. In order to keep the model parsimonious and interpretable, this model does not distinguish by the dose number that is missed (1st dose versus 2nd or 3rd dose); that is, the model can track changes in vaccine doses administered rather than changes in vaccination status of eligible individuals.

## 5. Conclusions

Vaccine administration rates of routine HPV vaccines decreased significantly among Swiss and Greek adolescents during the COVID-19 pandemic. These results show that the projected cumulative deficit of missed doses was substantial, totaling −24% and −22% in Switzerland and Greece, respectively. Nevertheless, with the implementation of tailored catch-up initiatives and a 6–13% increase in pre-pandemic vaccination rates, the HPV vaccination deficit could be eliminated by the end of 2025. Thus, it is important to highlight HPV immunization as a public health priority and support significant high-intensity catch-up initiatives to reverse the deficit and prevent long-term public health and economic consequences.

## Figures and Tables

**Table 1:** Cumulative deficit of HPV vaccine doses missed during the COVID-19 pandemic.

	Switzerland	Greece
Accumulated deficit of doses at start of the transition period	NA ^1^	27,527
Accumulated deficit of doses at start of the catch-up period	42,680	31,000
Accumulated deficit at start of the catch-up period as % of total annual doses administered during 2019	24.4%	21.7%
Accumulated deficit as a function of the number of months of 2019 ^2^	2.9	2.6

NA: not applicable. ^1^ A gradual increase in the number of vaccine doses disseminated in Switzerland was observed already in 2021 and assumed to be continued until the start of the catch-up period ^2^ Accumulated deficit at start of catch-up period as % of total annual doses administered during 2019 × 12 months.

**Table 2:** Catch-up rates required to clear the cumulative deficit of missed doses by country.

	Switzerland	Greece
**Base case: End of 2024**		
Additional number of HPV doses per month that must be administered compared to 2019	1226	975
Catch-up as % of total annual doses administered during 2019	8.4%	8.2%
**Optimistic scenario: End of 2023**		
Additional number of HPV doses per month that must be administered compared to 2019	1840	1545
Catch-up as % of total annual doses administered during 2019	12.6%	13%
**Pessimistic scenario: End of 2025**		
Additional number of HPV doses per month that must be administered compared to 2019	930	715
Catch-up as % of total annual doses administered during 2019	6.3%	6.0%

HPV: human papillomavirus.

## Data Availability

Data analyzed in this study have been shown in the article.

## Supplementary Materials

The following supporting information can be downloaded at: https://www.mdpi.com/article/10.3390/vaccines11020258/s1, Table S1. Model parameters by country; Figure S1. Relative reduction in the number of vaccine doses administered in 2020/2021; Figure S2. Deficits in vaccine doses administered due to the COVID-19 pandemic and model predictions for clearing the cumulative HPV deficit by the end of 2025 (pessimistic scenario); Figure S3. Deficits in vaccine doses administered due to the COVID-19 pandemic and model predictions for clearing the cumulative HPV deficit by the end of 2023 (optimistic scenario).
