# Supplementary material for: Impact of the COVID-19 Pandemic on HPV Vaccinations in Switzerland and Greece: Road to Recovery
Source: Vaccines (Basel). 2023 Jan 25;11(2):258. doi: 10.3390/vaccines11020258 (PMC9964352; doi:10.3390/vaccines11020258)
Supplement: Supplementary file 1 [file vaccines-11-00258-s001.zip › vaccines-2116432-supplementary.pdf]

vaccines

MDPI

Supplementary data

Table S1. Model parameters by country

	Switzerland	Greece
Data source	IQVIA data [24]	Data source
Targeted population	Girls and boys	Girls
Average doses per month during reference year (2019)	14,596	11,886
**Covid-19 related periods**		
Start of the COVID-19 period	03/2020	03/2020
Transition period	01/2021[1]	03/2022
Start of catch-up period	03/2022	09/2022

[1] A gradual increase in the number of vaccine doses disseminated in Switzerland was already observed in 2021 and was assumed to continue until the start of the catch-up period.

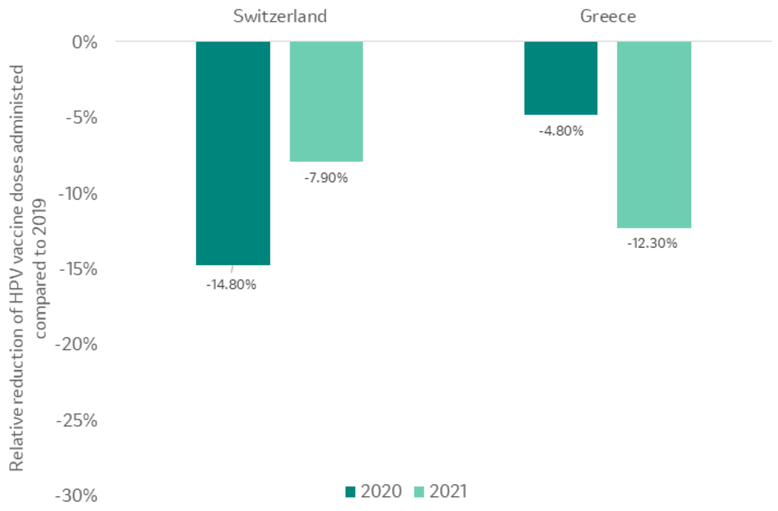

Figure S1. Relative reduction in the number of vaccine doses administered in 2020/2021

(**a**)

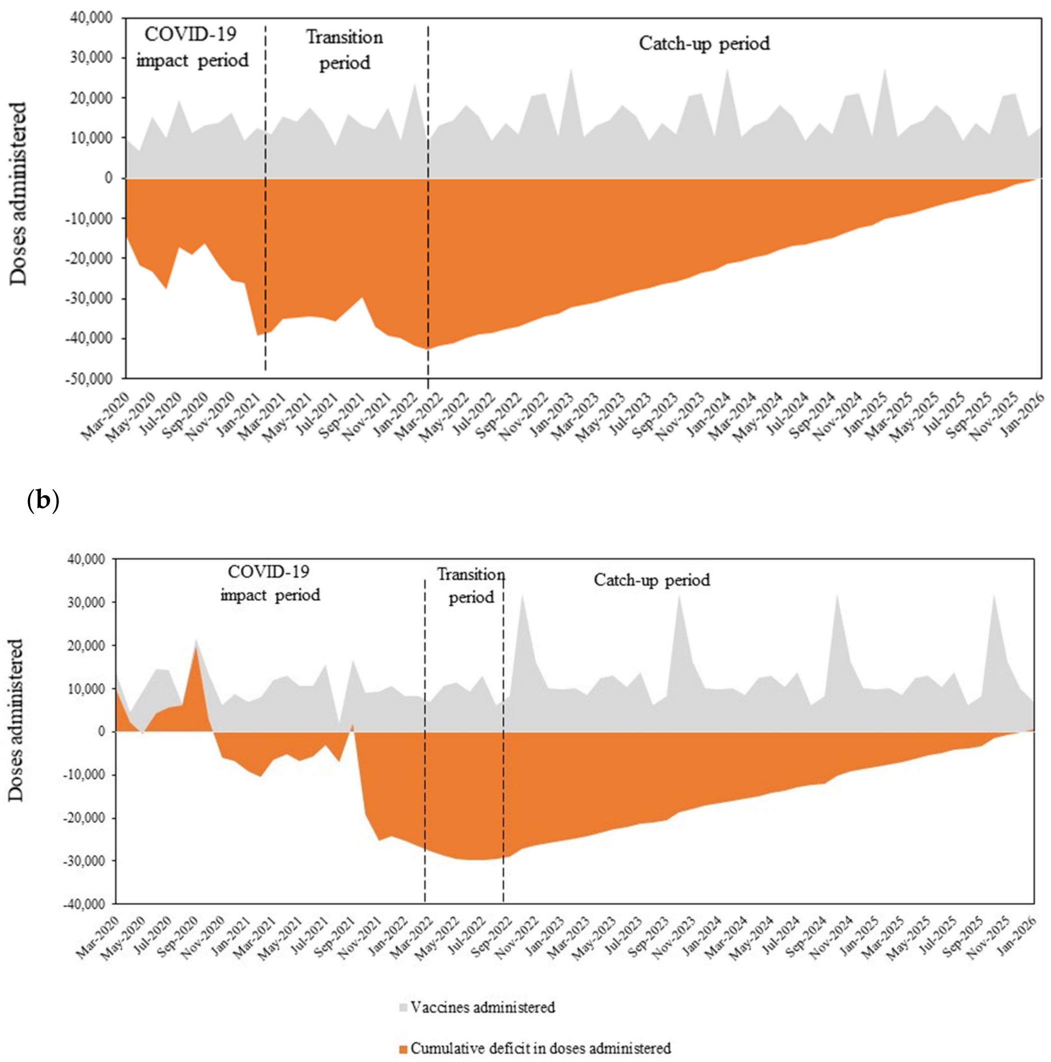

Figure S2. Deficits in vaccine doses administered due to the COVID-19 pandemic and model predictions for clearing the cumulative HPV deficit by the end of 2025 (pessimistic scenario) (a) Switzerland and (b) Greece.

(**a**)

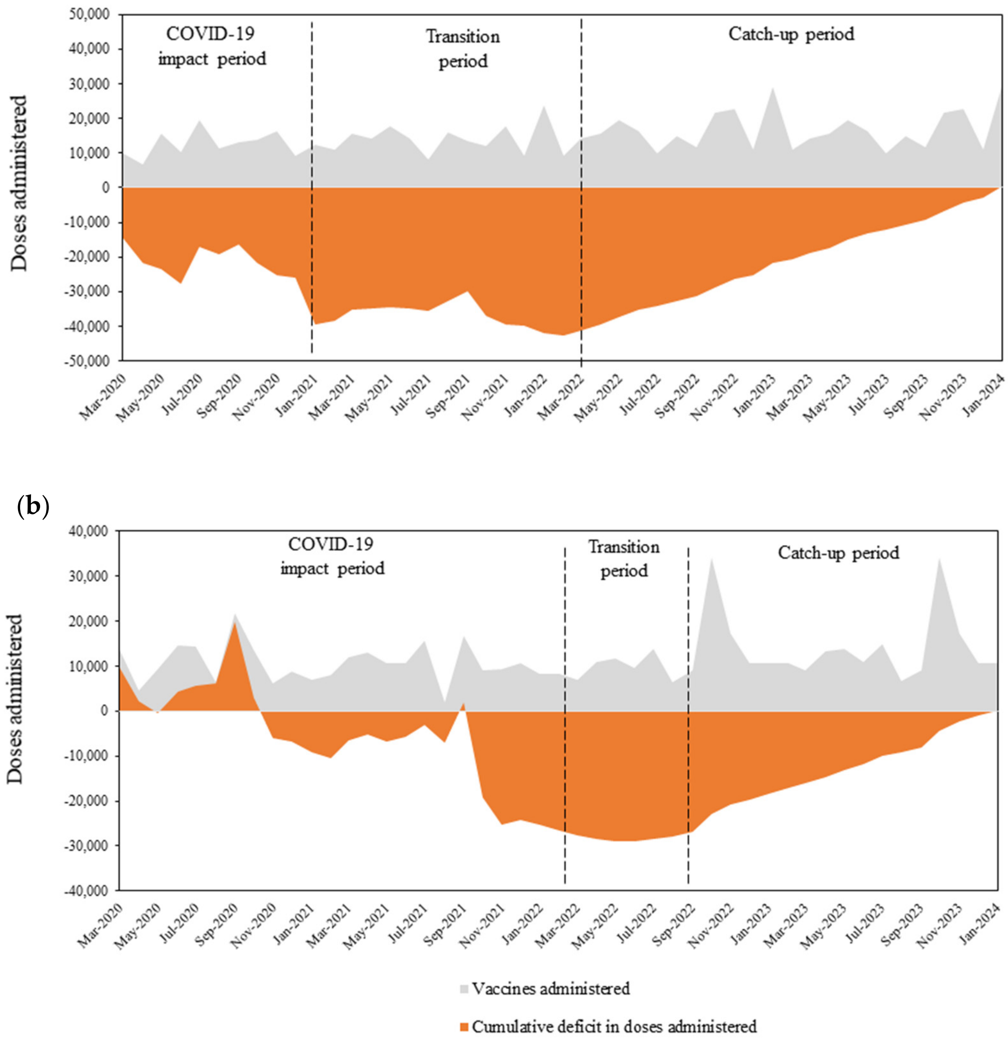

Figure S3. Deficits in vaccine doses administered due to the COVID-19 pandemic and model predictions for clearing the cumulative HPV deficit by the end of 2023 (optimistic scenario) (a) Switzerland and (b) Greece.
